# The Effect of Calcium and Iron (III) Oxides on Lead Spent Plates: Spectroscopic, Voltametric, and EIS Investigations

**DOI:** 10.3390/ma17174229

**Published:** 2024-08-27

**Authors:** Delia N. Piscoiu, Simona Rada, Sergiu Macavei, Adriana Popa, Claudia A. Crisan, Horatiu Vermesan, Eugen Culea

**Affiliations:** 1 Faculty of Materials and Environmental Engineering, Technical University of Cluj-Napoca, 400641 Cluj-Napoca, Romania; 2 National Institute for Research and Development of Isotopic and Molecular Technologies, 400293 Cluj-Napoca, Romania

**Keywords:** CaO-Fe_2_O_3_-Pb, spent anode, electrode, XRD, FTIR, UV–vis, EPR, EIS, CV, SLV

## Abstract

In this study, xCaO‧5Fe_2_O_3_‧(95−x)Pb glasses and vitroceramics containing various concentrations of calcium ions (from 0 to 50 mol% CaO) were prepared using the spent anodic plate of a car battery. X-ray diffraction analysis revealed changes in the network structure as a function of CaO content. The intensities of the IR bands due to the sulfate and sulfite units were lowered, indicating a decrease in the sulfurization degree within the lead network. In the UV–vis spectra, the presence of electronic transitions of the Fe^3+^, Pb^2+^, and Fe^2+^ ions were identified. The EPR spectra were characterized by resonance signals centered at about g ~ 2 and 4.3, corresponding to the trivalent iron ions. For the samples with 5 ≤ x ≤ 12, the signals decreased abruptly, suggesting a Fe^3+^→Fe^2+^ interconversion and the formation of the Fe_3_O_4_ crystalline phase. A considerable increase in the intensity of the signal centered around g ~ 2 was observed as the CaO concentration increased to 30% in the host matrix. Our results confirm that the higher CaO levels of 3 mol% are responsible for the increase in the radius of curvature of the semicircle arcs in the EIS plots and the decrease in their conductivity.

## 1. Introduction

The main purpose of waste management is to conserve resources and to protect the environment and human health [1]. According to the 2006 EU Directive, it is important to recycle a battery at the end of its life. Lead acid batteries are among the oldest and most widely used types of battery in the world. They are still used in the automotive industry and in energy storage systems [2,3,4]. Due to the increasing number of automobiles, demand for these types of batteries has grown such that they now comprise 79% of global battery consumption [5,6].

Used lead acid batteries are classified as hazardous waste and as secondary resources for recycling lead [7]. The recycling of batteries is highly efficient because it is estimated that 90% of used batteries are returned. In general, countries that manufacture batteries also have well-established management systems [8]. Lead production proves that there is an inseparable link between the manufacturing and recycling of car batteries.

The recycling of spent electrodes from car batteries is of major concern due to the importance of minimizing their negative effect on the environment and the secondary resources obtained from battery components.

The proper management and recovery of toxic materials from used car batteries leads to a lower pollution risk, as well as a clean and protected environment. The development of rechargeable batteries with minimal environmental impact is a critical issue as it aims to select chemicals with a minimal environmental footprint that will make recycling and integration into a circular economy as easy and affordable as possible.

The main recycling technologies are pyrometallurgy and hydrometallurgy. Their practical applications result in serious issues including random disposal and dumping of acid waste [9].

Lead is a toxic heavy metal in the environment and, as a result, is a source of concern in the spent car battery recycling industry. As a result, many issues that can arise during the recycling process have been pointed out. Incorporating spent electrodes from automotive batteries into a new life cycle of material used must be the first objective of recycling technology and hazardous waste management.

The melt quenching method was the first (and for a long time, the only) technique for making glass, and it is still the most important method for achieving industrial-scale glazing. The method is characterized by a versatile and very cost-effective process for obtaining materials with unique properties. Due to its ability to obtain amorphous materials in a relatively brief amount of time, the method is a valuable tool in environmental engineering and a sustainable circular economy [10,11].

The goals of the current work align with the interests of a sustainable industry, which, by exploiting various materials, can lead to the development of new directions for electrodes and offer a possible perspective on rechargeable batteries.

The aim of the research conducted in this paper was to prepare new materials from the anodic plates of a decommissioned automotive battery and investigate how they are affected by doping with calcium oxide and iron trioxide powder. Using the research methodologies outlined below, we facilitated the determination of the structural features of the prepared materials, as well as the evaluation of their electrochemical characteristics for prospective application as a novel electrode in a new battery. In this paper, the spent lead electrode of a car battery was used as a source of lead in the vitreous system with a xCaO∙5Fe_2_O_3_∙(95−x)Pb composition. We found that the spent electrode contains lead sulfate and can be continuously recycled without a great loss of performance, while also minimizing the release of hazardous substances into the environment, harnessing urban minerals, and decreasing solid waste production, including discarded batteries [11]. Calcium oxide is well known for its ability to absorb heavy metals [11,12,13,14], and iron trioxide has a low melting point. Therefore, the aim of this paper was to achieve the partial desulfurization of a spent electrode by adding a suitable amount of CaO and Fe_2_O_3_.

## 2. Experimental Procedure

### 2.1. Materials

Samples with the xCaO·5Fe_2_O_3_·(95−x)Pb composition, where x = 0, 3, 5, 8, 10, 12, 15, 20, 30, 40, and 50 mol% CaO were prepared according to the melt quenching method using the anodic plates of a car battery as a lead source and CaO and Fe_2_O_3_ powders (Sigma Aldrich, St. Louis, MO, USA).

The reagents were weighed in stoichiometric quantities on an analytical balance with four decimal places (0.0001 g) and mixed finely using an agate mortar. The obtained mixture was added to a ceramic crucible and then placed in an electric oven heated at 1050 °C. After ten minutes, the melted mixture was quickly inverted directly onto a stainless-steel plate at room temperature.

### 2.2. Methods

The crystalline or amorphous nature of the samples was studied via analysis of X-ray diffraction using a Rigaku diffractometer (Rigaku Corporation, Tokyo, Japan), with a Cu–Kα radiation wavelength of λ = 1.54 Å.

The FT–IR and UV–vis spectra of the samples were recorded using a JASCO FTIR 6200 spectrometer (JASCO, Tokyo, Japan) and a Perkin-Elmer Lambda 45 UV–vis spectrometer (Perkin-Elmer Corporation, Waltham, MA, USA), respectively, equipped with an integrating sphere and using the KBr pellet technique. IR and UV–vis spectra were recorded on pressed discs containing a mixture of sample and KBr in a proportion of 1:150. The EPR spectra were registered using a Bruker ELEXSYS 500 X-band spectrometer (Bruker Corporation, Billerica, MA, USA).

Electrochemical measurements, including cyclic voltammetry, sweep linear voltammetry, and spectroscopy of electrochemical impedance, were recorded using a PGSTAT 302N potentiostat/galvanostat and NOVA 1.11 software (Metrohm–Autolab B. V, Ultrecht, The Netherlands) using a scan rate of 10 mV/s.

The electrochemical cell used in the simulation consisted of three electrodes made of different materials, namely, recycled material doped with Fe_2_O_3_ and CaO as the working electrode, calomel as the reference electrode, and platinum as the counter electrode. The electrolyte solution was a sulfuric acid solution, 5M.

## 3. Results and Discussion

### 3.1. Analysis of X-ray Diffraction (XRD)

**Figure 1:**
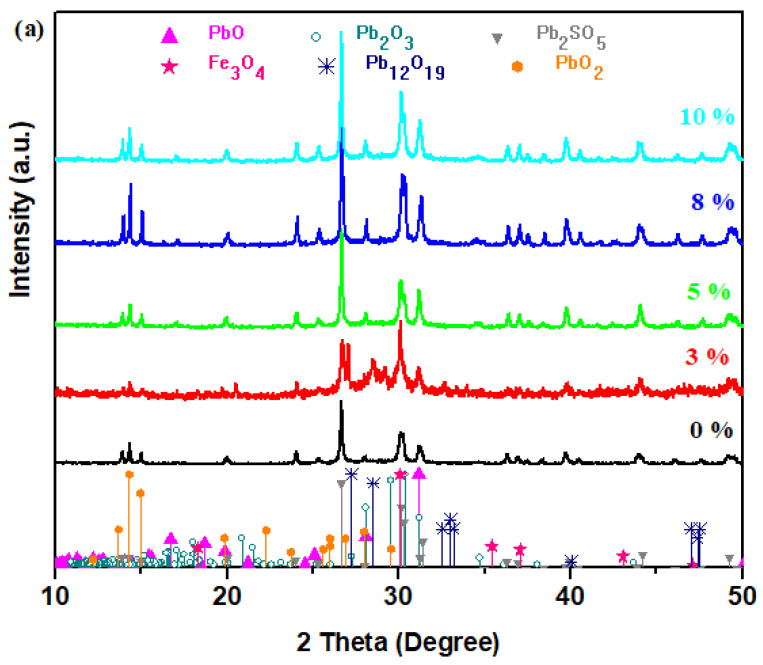

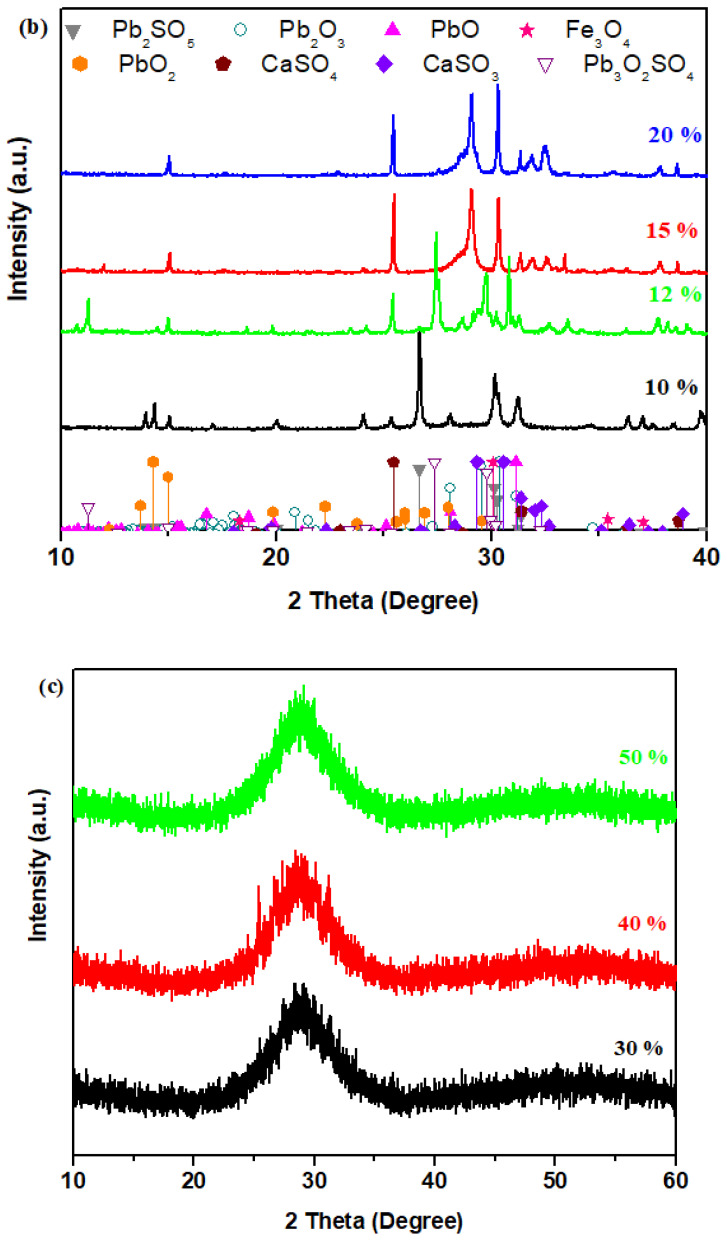
XRD diffractograms of xCaO·5Fe_2_O_3_·(95−x)Pb samples: (**a**) x = 0, 3, 5, 8, and 10 mol%; (**b**) x = 12, 15, 20 mol%; (**c**) x = 30, 40, and 50 mol% CaO.

X-ray diffraction analysis is used to determine the structure, types of atoms in the crystalline state, angles, and interatomic distances. Figure 1 represents the X-ray patterns of the vitreous system in the xCaO·5Fe_2_O_3_·(95−x)Pb composition, where x = 0–50 mol% CaO.

The XRD analysis shows in the host matrix a vitroceramic structure with varied Pb_2_SO_5_, Fe_3_O_4_, PbO, and PbO_2_ crystalline phases. By adding the CaO powder to a content of up to 20 mol%, the presence of varied crystalline phases was found in glass–ceramics.

By doping with 3% CaO, the presence of the Pb_2_SO_5_, Pb_12_O_19_, PbO, Fe_3_O_4,_ and Pb_2_O_3_ crystalline phases was detected. The intensity of the characteristic peaks of the Pb_2_SO_5_ phase was also decreased.

For samples with 5 ≤ x ≤ 10 mol% CaO, the intensity of the diffraction peaks due to the Pb_12_O_19_ (Pb_12_O_19_ ≡ 5PbO·7PbO_2_) and Pb_2_O_3_ crystalline phases disappears and the content of the PbO_2_ and Pb_2_SO_5_ oxosulfated phases increases. The intensities of the main diffraction peaks assigned to the Fe_3_O_4_ and PbO crystalline phases are intensified for the samples with 8 and 10 mol% CaO.

The increase in CaO content up to 12% shows the appearance of the new oxosulfated phases of the lead, namely, the Pb_3_O_2_SO_4_ phase with monoclinic structure as the major phase, and the Pb_2_SO_5_ crystalline phase was not evidenced. In addition, glass–ceramics also have the CaSO_4_, CaSO_3_, PbO, and Fe_3_O_4_ crystalline phases with a rhombohedral structure.

By increasing the CaO content, between 15 ≤ x ≤ 20 mol%, the oxosulfated phases of lead disappeared from the structure of the vitroceramic due to the improvement amount of the CaSO_4_ phase and the appearance of the CaSO_3_ phase with monoclinic structure. Other crystalline phases of these glass–ceramics are Pb_2_O_3_, Fe_3_O_4_, and, in smaller quantities, the PbO crystalline phase.

For the levels of 15 and 20% CaO, a total desulfurization process of the sulfate lead was achieved because the content of oxo-sulfated lead phases is below the detection limit of the diffractometer.

The addition of higher CaO contents of up to 50% CaO indicates the formation of an amorphous structure (as shown in Figure 1c). Through desulfurization of the host matrix, the lead ions can participate as a network formed with oxygen ions by the creation of [PbO_4_] structural units. The intensity of the IR bands situated between 800 and 980 cm^−1^ was enriched in accordance with the IR data presented in Section 3.2. This region is assigned to the Pb-O stretching vibrations in the [PbO_n_] structural units, where *n*= 3, 4, and 6.

### 3.2. Infrared Spectra

**Figure 2:**
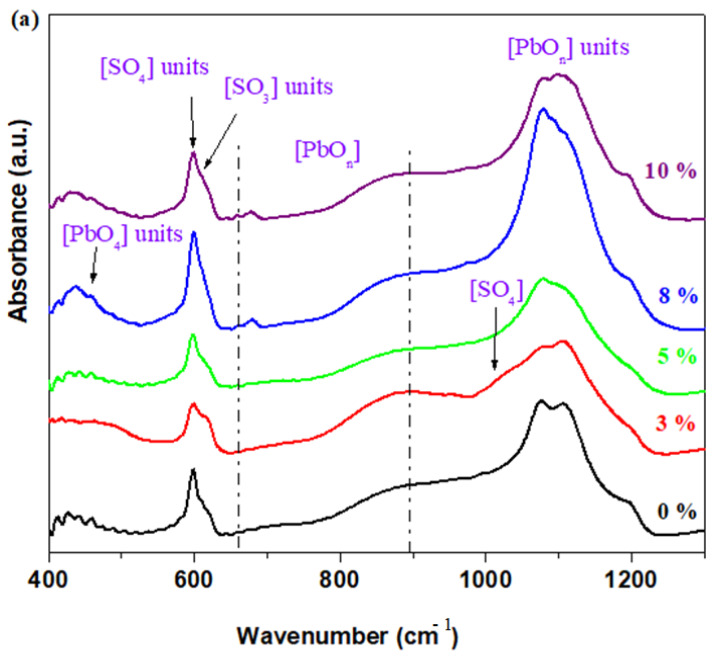

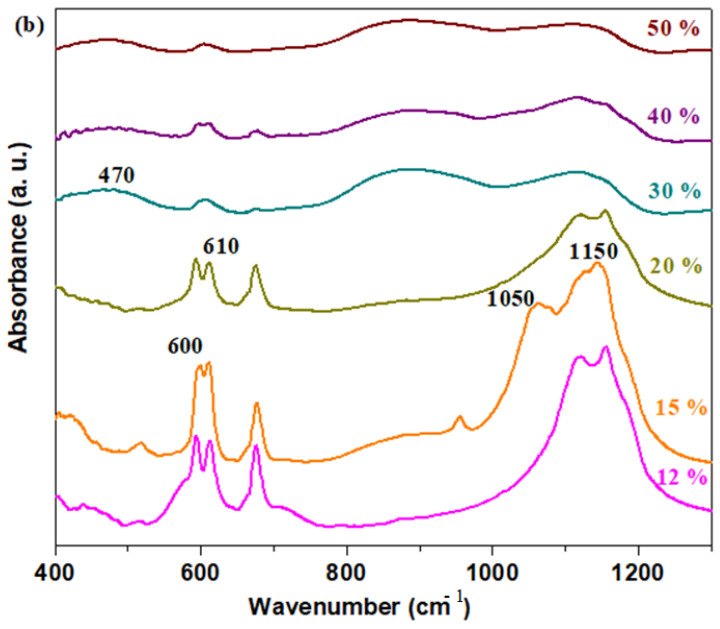
FTIR spectra of xCaO·5Fe_2_O_3_·(95−x)Pb samples: (**a**) x = 0 10 mol%; (**b**) x = 1250 mol% CaO.

The FTIR spectra of the prepared vitreous system are shown in Figure 2. In this analysis, several structural modifications of the band intensity can be seen. This corresponds to the different structural units obtained by doping the host matrix with different quantities of calcium oxide.

The first region of IR bands, located between 375 and 575 cm^−1^, corresponds to the superposition of elongation vibrations of the Ca-O, Fe-O bonds with deformation vibrations of the Pb-O-Pb angles (at 470 cm^−1^) in the [PbO_4_] structural units [15]. The intensity of the IR band centered at 470 cm^−1^ reaches maximum values for samples doped with high CaO contents (for samples with 30 ≤ x ≤ 50% CaO).

The second range of mid-intensity IR bands between 575 and 675 cm^−1^ can be associated with the S-O elongation vibrations of the sulfate units (at 600 cm^−1^) and sulfite units (at 610 cm^−1^). The oxosulfated lead phase can be evidenced in the IR spectrum via the presence of S-O stretching vibrations in the sulfate units. The IR bands located at 600, 1050, and 1150 cm^−1^ are assigned to the elongation vibrations of the S-O bonds in the sulfate units, and their intensities can be decreased through doping [12]. The number of sulfate and sulfite units decreases through doping, with higher CaO levels. The dopant plays a main role in the break down of the oxosulfated phases involving lead ions.

By doping with 3% CaO content, the intensity of the IR band centered at about 600 cm^−1^ was minimized, indicating that the amount of oxosulfated phases decreased, confirming the XRD data.

The intensity of the two IR bands corresponding to the sulfate and sulfite units increased gradually for samples with 12 ≤ x ≤ 20 mol% CaO and decreased at higher dopant levels. For samples with 12 ≤ x ≤ 20 mol% CaO, a new, well-formed IR band appears at about 670 cm^−1^ due to non-symmetric elongation vibrations of the Pb-O bond in the [PbO_n_] structural units.

At higher CaO contents (up to 12%), the formation of Pb_3_O_2_SO_4_ ≡ 2PbO·PbSO_4_ oxosulfated phases was evidenced, in accordance with the XRD data, due to excess oxygen atoms.

Samples doped with x = 15 and 20 mol% CaO do not have the oxosulfated phase of lead because calcium ions substituted the lead ions in Pb_2_SO_5_ according to the chemical reaction presented in Equation (1):

CaO + PbO·PbSO_4_→CaSO_4_ + PbO_2_ + Pb. (1)

The peaks situated at intensities below 675 cm^−1^ were modified nonmonotonically with x due to the conversion of oxosulfated lead into calcium sulfate and sulfite phases. The lead oxide from the lead oxosulfated phase readily forms other lead oxides in the presence of excess oxygen.

The domain of intense IR bands situated between 770 and 1250 cm^−1^ could be due to the overlapping vibrations of the sulfate, i.e., sulfite units with [PbO_n_] units with *n* = 3, 4, and 6. The IR band centered at about 855 cm^−1^ could be also due to the stretching vibrations of the Ca-O bonds [16]. The intensity of these bands reaches a maximum value for the CaO levels of at least 30%. These evolutions suggest the conversion of the lead network into calcium–lead glasses.

The CaO and Fe_2_O_3_ have a role in the desulfurization of spent plates. Fe_2_O_3_ reduces the synthesis temperature and can be a network former or modifier. The effect of calcium oxide on the lead spent plates is of network modifier.

### 3.3. Ultraviolet–Visible (UV–Vis) Spectra: Optical Gap Energy

Mathilde Patin et al. [8] described the significant importance of Fe^3+^ as the most relevant metal ion responsible for the UV absorption of glasses and vitroceramics.

**Figure 3:**
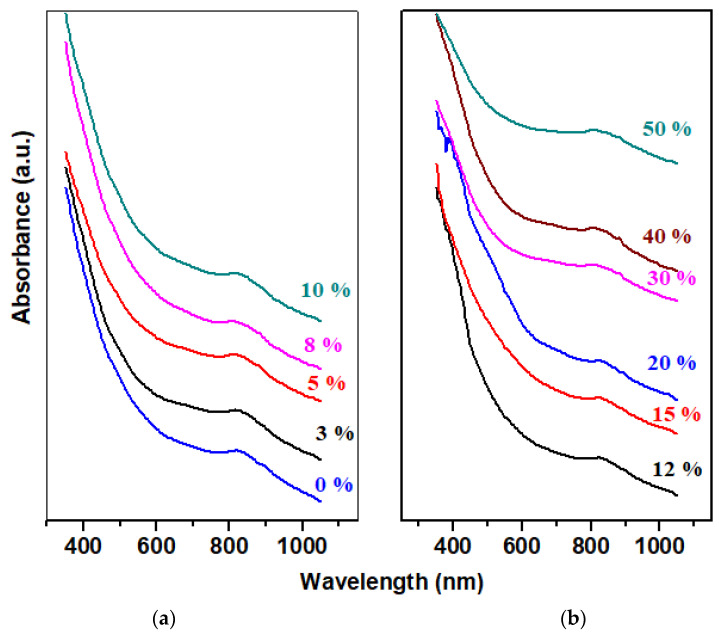
UV–vis spectra of xCaO·5Fe_2_O_3_·(95−x)Pb samples: (**a**) x = 0–10 mol%; (**b**) x = 12–50 mol% CaO.

The UV–vis spectra of the xCaO‧5Fe_2_O_3_‧(95−x)Pb system are presented in Figure 3.

The intensity of the half band centered at 310 nm, attributed to Pb^2+^ ions [17,18], decreases with the addition of CaO contents above 30%. This evolution is due to the conversion of Pb^2+^ to Pb^4+^ ions and the increase in polymerization degree.

For samples with x ≤ 5%, a decrease in intensity of the UV–vis half-band centered at 400 nm and an increase in the intensity of the UV–vis bands located in the region between 450 and 850 nm are observed [19]. These structural modifications show the conversion of the ionic positions of lead towards its role as a network former. The increase in intensity of the UV–vis bands centered between 550 and 750 nm indicates the electronic transitions of Fe^3+^ ions coordinated with oxygen. The d–d transitions near the infrared region can be attributed to the octahedral geometries of the divalent iron ions coordinated with oxygen.

The high-intensity band half-centered at 400 nm increases for the samples with 8 ≥ x ≤ 40% and decreases drastically for the sample with x = 50 mol% CaO. The intensity of the UV–vis bands between 500 and 750 nm increases through doping with higher concentrations of CaO.

**Figure 4:**
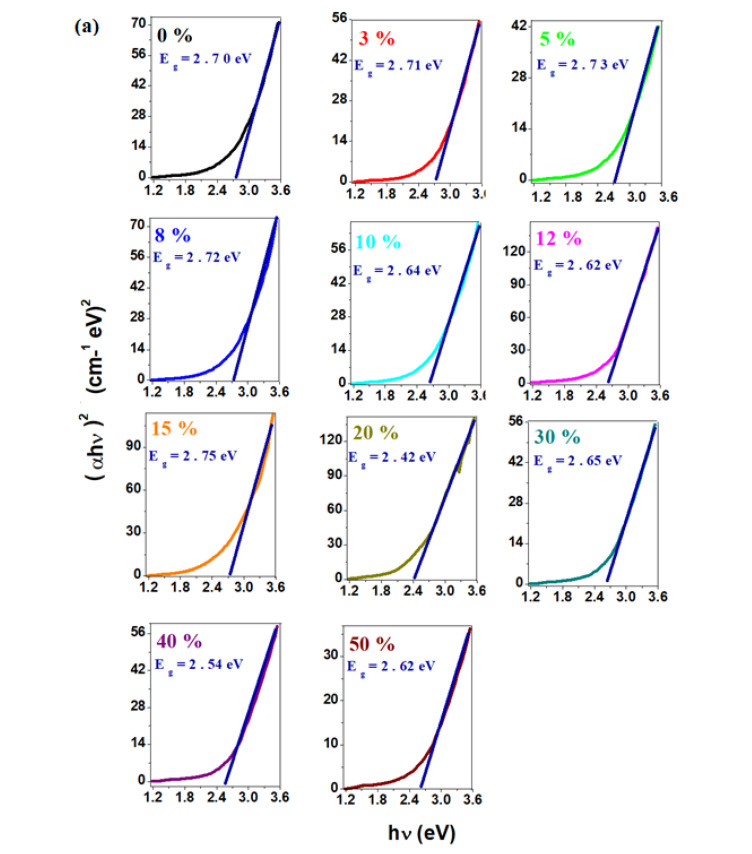

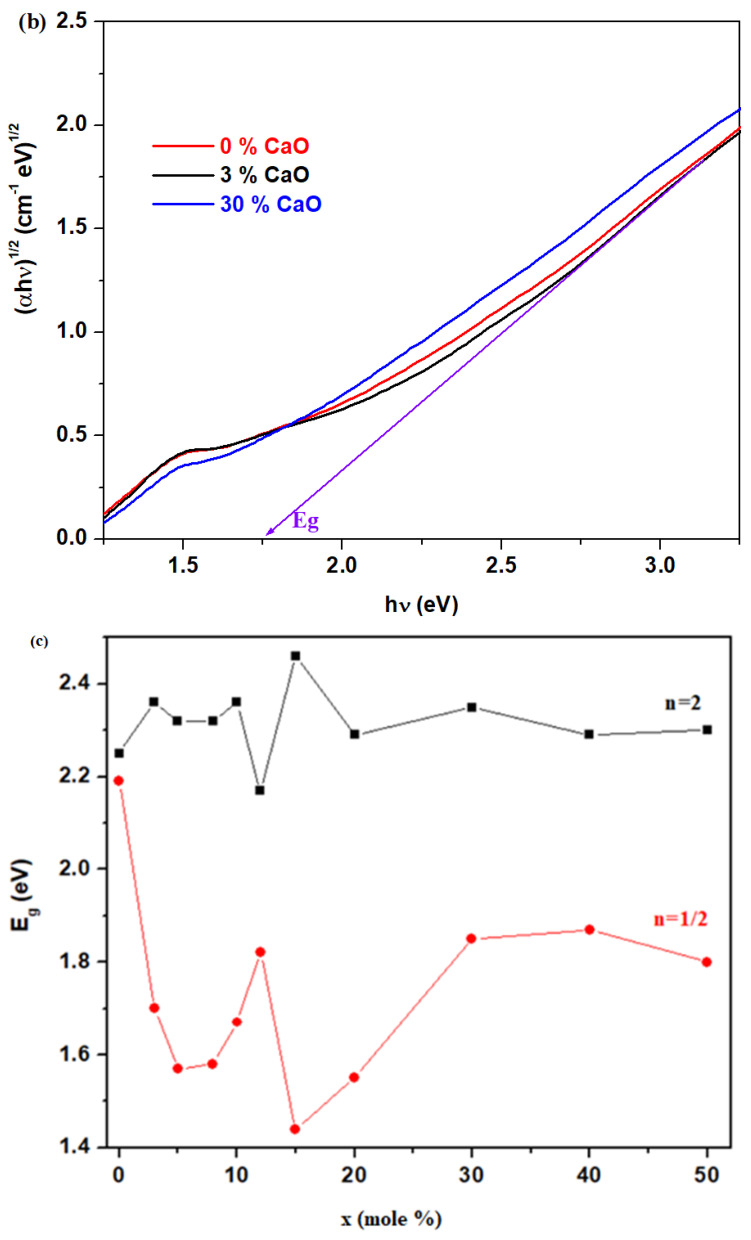
Dependence of (**a**) (αhν)^2^ and (**b**) (αhν)^1/2^ on *hν*. Extrapolation of the optical gap energy, *Eg*. (**c**) Compositional evolution of the optical gap energy values, *Eg*, on CaO content (x in mol%).

The graphical representations of the values of (αhν)^1/2^ and (αhν)^2^, respectively, as a function of photon energy, *hν*, and the optical gap energy values, *Eg*, for direct (*n* = 1/2) and indirect (*n* = 2) transitions of the studied systems, are shown in Figure 4.

The optical band gap values are between 2.42 eV and 2.75 eV, indicating semiconductor behavior [2,18,20]. In the case of direct transitions, the optical gap energy values range from 1.40 eV to 1.96 eV. The dependence of the optical gap energy values, *Eg*, on the composition of the recycled material (x mol% CaO) for direct and indirect transitions, is shown in Figure 4c. The sample with x = 15 mol% CaO has the lowest value of the gap energy.

### 3.4. Electron Paramagnetic Resonance (EPR) Spectra

**Figure 5:**
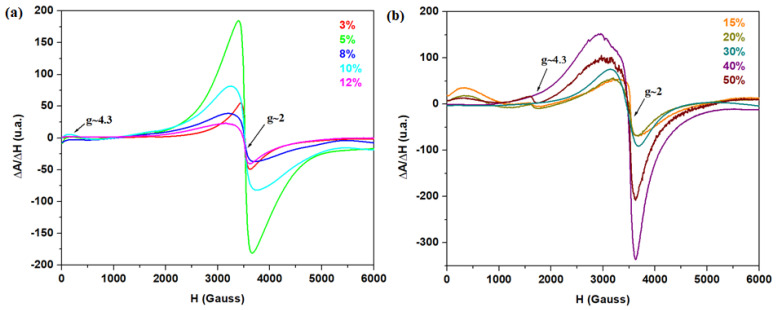
EPR spectra for the recycled system: (**a**) x = 0, 3, 5, 8, 10, and 12% CaO; (**b**) x = 15, 20, 30, 40, and 50% CaO.

Investigations via electronic paramagnetic resonance (EPR) spectroscopy are used to obtain information about paramagnetic centers, namely, iron ions [21]. The EPR spectra of the recycled system are presented in Figure 5. The EPR data show two values of the gyromagnetic factor, namely, g ~ 2 and g ~ 4.3, assigned to the Fe^3+^ ions, which depend on the CaO concentration in the vitreous system. The first resonance signal located at g ~ 2 is characterized by clustered Fe^3+^ ions or species involved in dipole–dipole interactions arranged in geometries with less distorted octahedral symmetry and low crystal fields [12,13]. The resonance signal located at g ~ 4.3 is attributed to isolated Fe^3+^ ions dispersed in distorted octahedral symmetries [21,22].

The resonance signal of low intensity situated at g ~ 4.3 increases slightly via doping with higher dopant levels (above 30% CaO). This increase is due to the high content of isolated Fe^3+^ ions located in octahedral sites.

The intensity of the peak situated at g ~ 2 attains maximum value for the sample with 5% CaO, decreases for samples with 8 and 12% CaO, and then increases again for adding 15% CaO. This decrease in the resonance signals corresponds to the Fe^3+^ ions and shows the conversion of Fe^3+^ into Fe^2+^ ions, as well as the formation of Fe_3_O_4_ and O^2+^·Fe^3+^_2_O_3_ crystalline phase, confirming the XRD data by increasing CaO content up to 12 mol%.

For x ≥ 30% CaO, the signal strengths situated at g ~ 2 and 4.3 increase with the addition of higher dopant levels in the host matrix. The iron ions are implied as a network formed in an amorphous structure.

### 3.5. Cyclic Voltammetry Measurements

Measurements of cyclic voltammetry, linear sweep voltammetry, and electrochemical impedance spectroscopy (EIS) will be used to demonstrate the electrochemical performance of materials prepared in the CaO-Fe_2_O_3_-Pb composition from spent anodic plates as new electrodes for the automobile battery. In cyclic voltammetry, the voltage applied to the circuit terminals varies between two potentials, one maximum positive and one maximum negative, with a constant gradient of variation [22].

**Figure 6:**
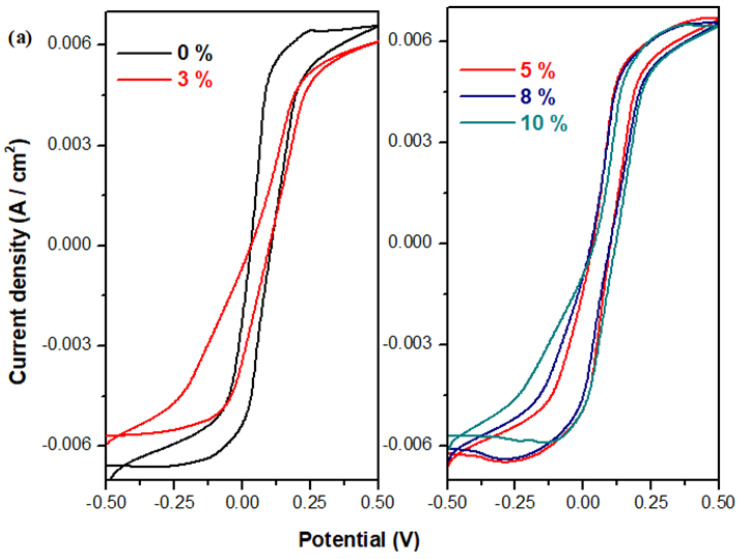

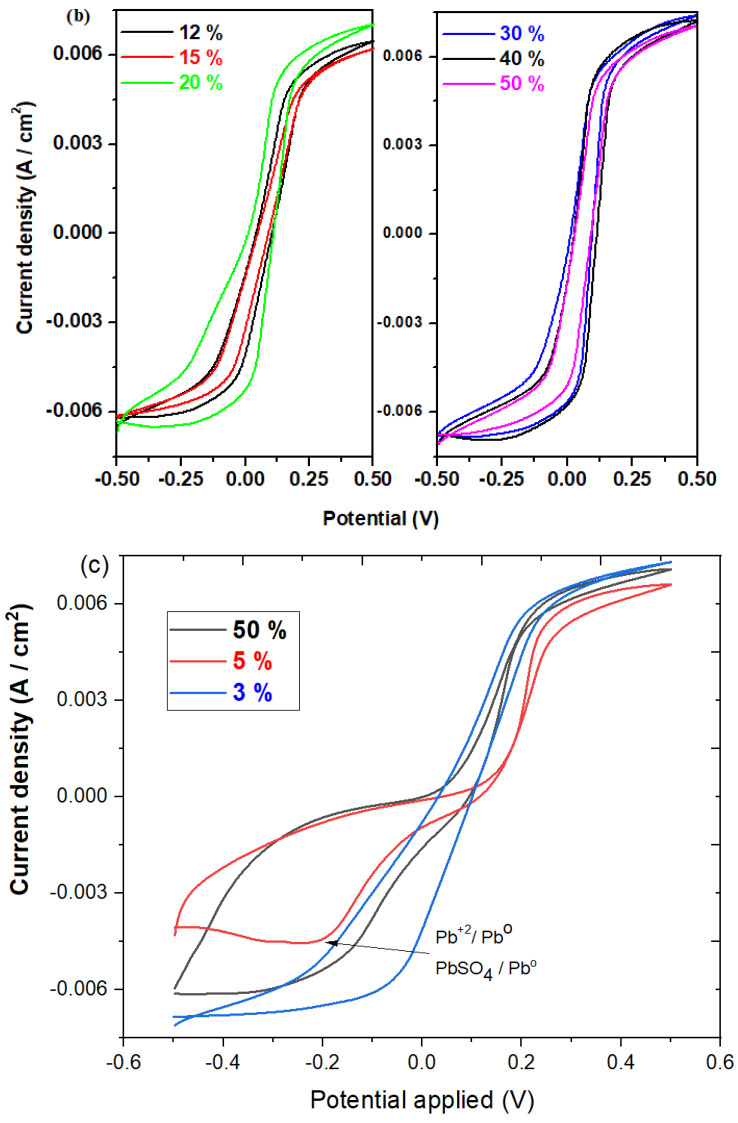
Cyclicvoltammograms scanned after one cycle of the electrode materials in the xCaO·5Fe_2_O_3_·(95−x)Pb composition: (**a**) x = 0–10% CaO; (**b**) x = 12–50% CaO; (**c**) x = 3, 5, and 50%CaO.

**Figure 7:**
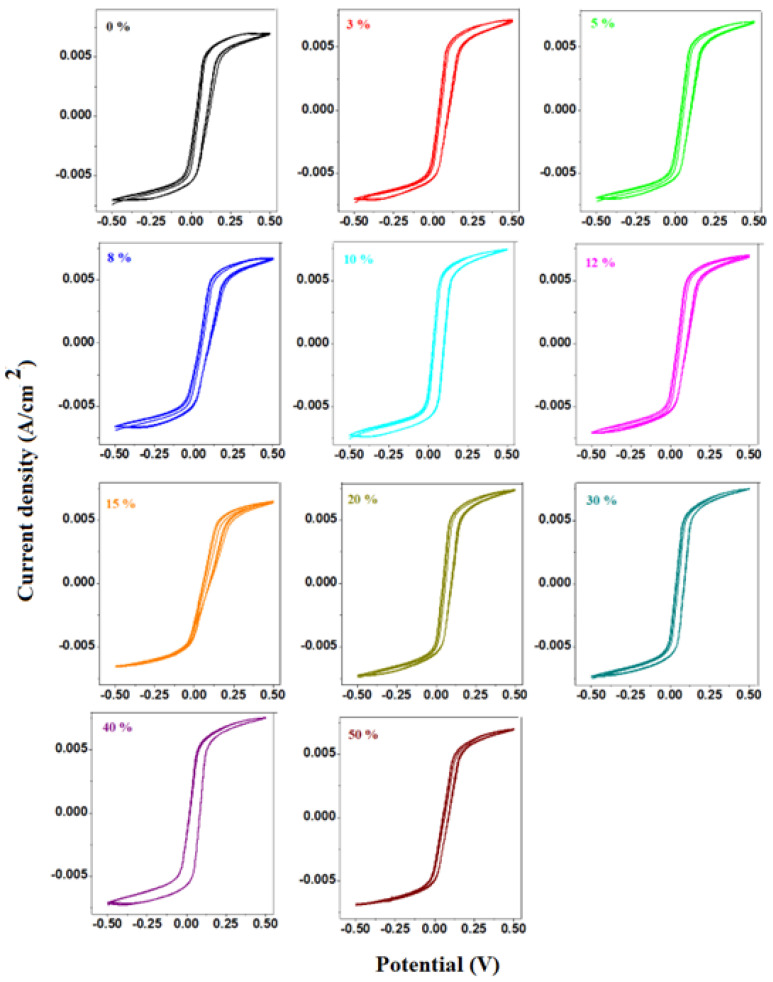
Cyclic voltammograms scanned after three cycles of studied vitreous system.

Cyclic voltammograms scanned after one cycle and three cycles for the prepared electrode materials are shown in Figure 6 and Figure 7.

In the cyclic voltammograms, the reduction peak appears at about −0.21 V and the oxidation peak seems to be situated at 0.2V. The cathodic peak corresponds to the overlapping of two waves, the first situated at −0.13 V, associated with Pb^2+^/Pb^0^ redox process, and the second at −0.356V due to the PbSO_4_/Pb system. The smaller anodic peak, centered at about 0.2V, can be observed in the sample with 0% CaO and can be assigned to PbO_3_^−2^/PbO_2_^−2^ redox process.

Furthermore, for the detailed studies regarding the electrochemical performances of the electrode materials, the linear sweep voltammograms and their electrochemical parameters were determined.

**Figure 8:**
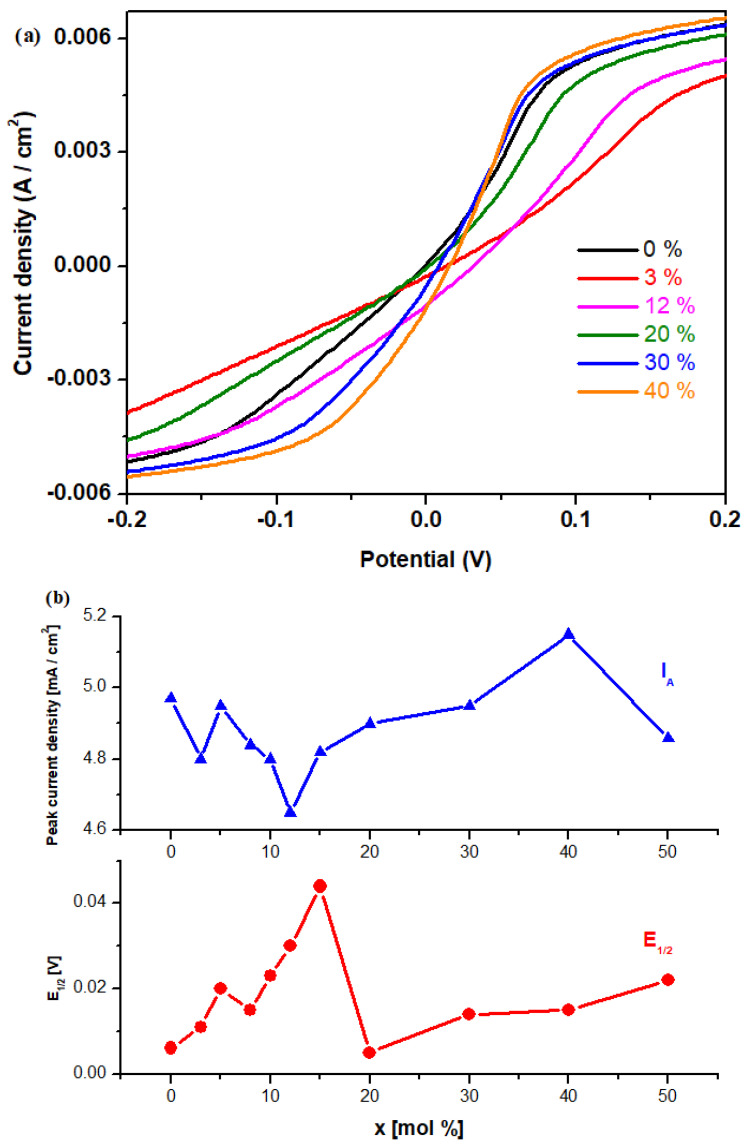
(**a**) Linear voltammograms of studied electrode materials. (**b**) Compositional evolution of the electrochemical parameters: half-wave potential (E_1/2_) and peak current density (I_A_).

Linear sweep voltammograms of the samples are represented in Figure 8a. The electrochemical parameters obtained via linear sweep voltammetry of the half-wave potential, E_1/2_, after scanning the first cycle, are listed in Table 1. Figure 8b shows the compositional evolution of some electrochemical parameters, namely, half-wave potential, E_1/2,_ and peak current density, I_A_. A decreasing trend of peak current density was observed at smaller CaO content concentrations, attaining a minimum value for the sample with 12 mol%. After that, by adding greater CaO concentrations in the host matrix, the peak current density increases slowly up to 40 mol% and then decreases again at 50 mol%.

The values of half-wave potential increase gradually up to 15 mol% and decrease for the sample with 20%. At higher dopant levels, an increasing trend can be observed to 50 mol% CaO.

The negative shift in the half-wave potential relates to the reversibility of the system. The samples with x = 0, 3, 8, 30, and 40 mol% CaO have smaller values of half-wave potential of approximately 0.1 V, which indicates a better reversibility of cyclic voltammetry for electrode material. For the 15% CaO sample, the half-wave potential has the highest value, indicating poor reversibility of the cyclic voltammogram.

### 3.6. Electrochemical Impedance Spectroscopy (EIS)

The EIS method indicates the performance of the studied battery. From the impedance spectra, an equivalent circuit is designed, and the significance of the various components is determined. An equivalent circuit is an electrochemical cell which is indicated by a network of resistors and capacitors [23]. In other words, the electrical circuit made up of physical elements can be assigned an impedance spectrum identical to that of the electrochemical system.

In this paper, the simplified equivalent circuit model includes the electrical bulk resistance and the internal resistance of charge transfer at the interface between electrode and electrolyte, R_ct_. Diffusion can create an impedance called a Warburg impedance, W. The polarization resistor, R_ct_, is connected in series with a bulk resistor, R_b_, and the Warburg impedance, W, and in parallel with the surface deposited double layer capacitor, C. The allocations of bulk resistances, R_b_, and resistance of charge transfer, R_ct_, are shown in Table 2.

**Figure 9:**
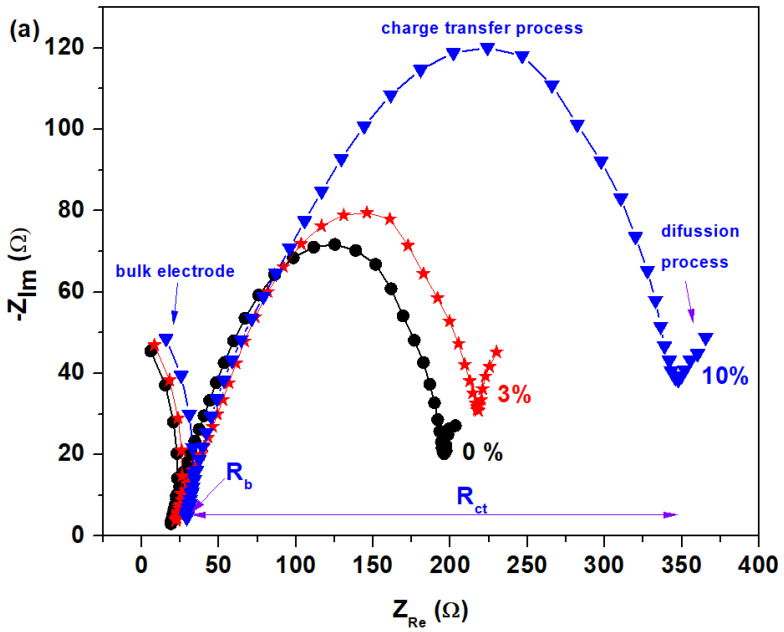

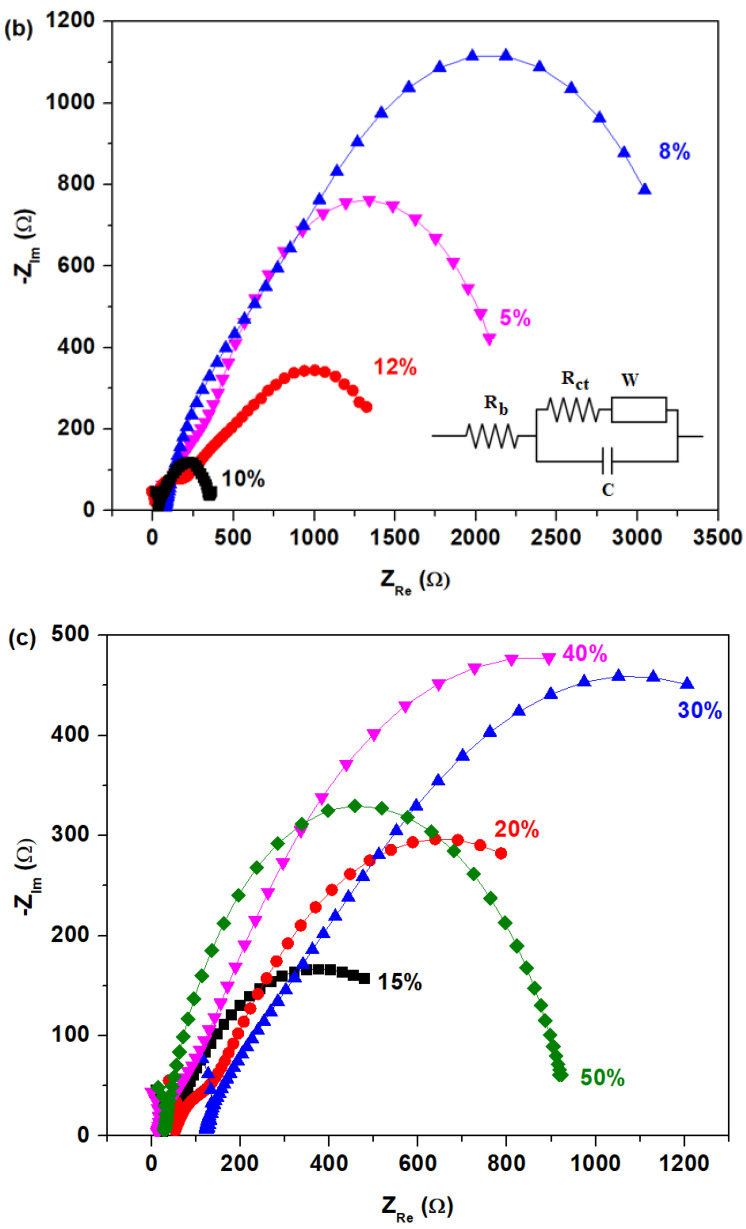

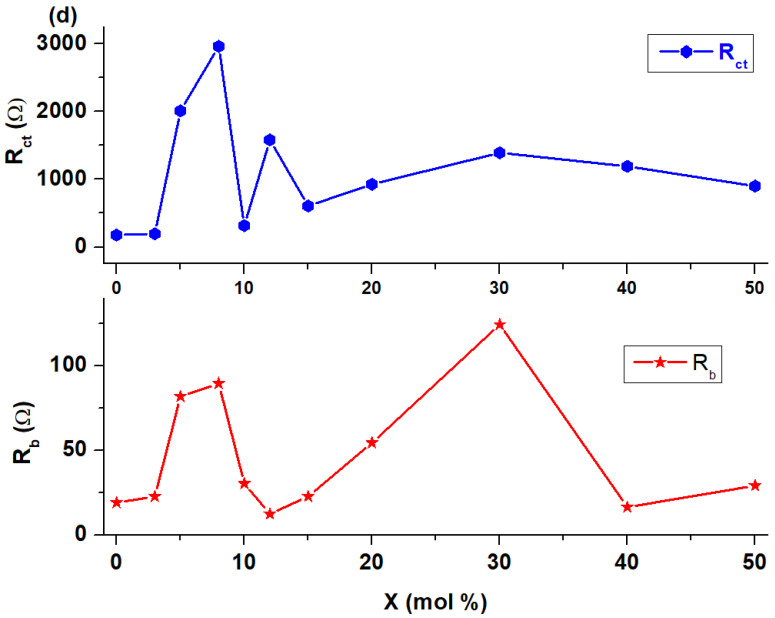
Nyquist diagrams of the complex impedance obtained at different concentrations for (**a**) x = 0, 3 and 10%, (**b**) x = 5–12%, and (**c**) x = 15–50%. (**d**) Compositional dependence of the bulk resistance (R_b_) of the sample.

The EIS spectra can be used to characterize the interfacial properties of the electrode [24]. Figure 9 represents Nyquist plots from EIS tests. The horizontal axis (real axis) represents the resistance, and the normal axis (imaginary axis) represents the capacitance of the equivalent circuit [25]. The impedance spectrum has a bulk arc situated at high frequency, a semicircle at medium frequency, and an electrode arc at low frequency [26]. All EIS spectra show semicircles, and the slant line is attributed to changes in the resistance and capacitance components of the electro/electrolyte interface. The first arc is attributed to the bulk electrical properties of the recycled material, the second arc shows the polarization effect of the electrode/sample, and the slant line presents diffusion control [27].

We also found that the CaO concentration levels have varied effects on the EIS plots. It can be observed in Figure 9b that the profile of the EIS curve can be divided into three regions, namely, high-, medium-, and low-frequency regions. The medium- and low-frequency regions correspond to the charge transfer process and diffusion, respectively. For example, the addition of 30 or 40% CaO can cause more modifications in the profile of the electrode EIS curve than that of 15%. For the samples with x = 30% and 40%, the charge transfer and diffusion regions will yield an increase in curvature radius of the semicircle arc in the EIS plot, which will increase the charge transfer impedance.

In the Nyquist curve, we can define the bulk resistance, *R_b_*, at the intersection of these arcs. Figure 9c indicates the modification of the bulk resistance, *R_b_*, and charge transfer resistance, *R_ct_*, at various doping levels.

For the samples with 0, 3, 12, 15, and 40% CaO, the impedance spectra moved to the left, and the bulk electrical resistance, *R_b_*, was decreased in comparison with its analogs. The decrease in *R_b_* value can be linked to the higher conductivity of the sample. The smaller values of the resistance to charge transfer were obtained for the samples with 0 and 3 mol% CaO.

By doping the host matrix with 30% CaO contents, the impedance spectra shift towards a lower frequency, and the values of the bulk resistance are gradually modified, reaching a maximum value. The semicircle diameter in the EIS curve is significantly larger than that of the other samples, suggesting a decrease in the conductivity of the electrode.

The results of this paper confirm that the samples with 0% CaO and 3% CaO have the lowest bulk resistance and charge transfer resistance values in the low-frequency region, making them progressively more sensitive to charge transfer and diffusion processes. The sample with 3% CaO has, in structure, several types of lead oxides and a smaller amount of Pb_2_SO_5_ crystalline phase when compared with the host matrix. In brief, this paper suggests that the sample with 3% CaO, recycled from the anodic plate of a car battery, could be used as a new electrode material for battery applications.

## 4. Conclusions

New vitreous materials in the xCaO‧5Fe_2_O_3_‧(95−x)Pb composition, where x = 0–50% CaO, were obtained at 1050 °C using as raw material the spent anode of a car battery.

The analysis of the X-ray diffractogram shows a vitroceramic structure with different crystalline phases at lower CaO contents and an amorphous structure at higher dopant levels.

IR data show that a process of accommodation with excess oxygen occurs through the decrease in sulfate and sulfite units.

Analysis of UV–vis data indicates the presence of Fe^3+^ and Pb^2+^ ions in the studied materials. The EPR data show signals attributed to Fe^3+^ ions in the varied coordinated geometries.

Electrochemical studies show that the electrode materials with x = 0 and 3 mol% CaO have good reversibility of cyclic voltammetry and smaller resistance values. The prepared materials have practical applications, including their use as a new electrode in lead-acid batteries.

## Figures and Tables

**Table 1:** Electrochemical parameters obtained via linear sweep voltammetry of the half-wave potential (E_1/2_) and peak current density after the scan of the first cycle.

x [mol %]	E_1/2_[V]	Peak Current Density, I_A_ [mA/cm^2^]
0%	0.006	4.97
3%	0.011	4.80
5%	0.020	4.95
8%	0.015	4.84
10%	0.023	4.80
12%	0.030	4.65
15%	0.044	4.82
20%	0.005	4.90
30%	0.014	4.95
40%	0.015	5.15
50%	0.022	4.86

**Table 2:** The allocation of bulk resistance (R_b_) and charge transfer resistance (R_ct_) for the studied samples.

x [mol %]	R_b_ [Ω]	R_ct_ [Ω]
0%	19.36	177
3%	22.72	194.3
5%	81.7	2009.3
8%	89.42	2962.5
10%	30.31	314.69
12%	12.3	1581
15%	22.73	605.27
20%	54.35	925
30%	124.2	1391
40%	16.42	1190.2
50%	29.05	898.9

## Data Availability

All data is contained within the article.
